# Ethical layering in AI-driven polygenic risk scores—New complexities, new challenges

**DOI:** 10.3389/fgene.2023.1098439

**Published:** 2023-01-26

**Authors:** Marie-Christine Fritzsche, Kaya Akyüz, Mónica Cano Abadía, Stuart McLennan, Pekka Marttinen, Michaela Th. Mayrhofer, Alena M. Buyx

**Affiliations:** ^1^ Institute of History and Ethics in Medicine, TUM School of Medicine, Technical University of Munich, Munich, Germany; ^2^ Department of Science, Technology and Society (STS), School of Social Sciences and Technology, Technical University of Munich, Munich, Germany; ^3^ Biobanking and Biomolecular Resources Research Infrastructure Consortium - European Research Infrastructure Consortium (BBMRI-ERIC), Graz, Austria; ^4^ Department of Science and Technology Studies, University of Vienna, Vienna, Austria; ^5^ Helsinki Institute for Information Technology HIIT, Aalto University, Helsinki, Finland

**Keywords:** genomics, polygenic risk score, deep neural network (DNN), machine learning (ML), artificial intelligence–AI, stratification, predictive medicine, ethical

## Abstract

Researchers aim to develop polygenic risk scores as a tool to prevent and more effectively treat serious diseases, disorders and conditions such as breast cancer, type 2 diabetes mellitus and coronary heart disease. Recently, machine learning techniques, in particular deep neural networks, have been increasingly developed to create polygenic risk scores using electronic health records as well as genomic and other health data. While the use of artificial intelligence for polygenic risk scores may enable greater accuracy, performance and prediction, it also presents a range of increasingly complex ethical challenges. The ethical and social issues of many polygenic risk score applications in medicine have been widely discussed. However, in the literature and in practice, the ethical implications of their confluence with the use of artificial intelligence have not yet been sufficiently considered. Based on a comprehensive review of the existing literature, we argue that this stands in need of urgent consideration for research and subsequent translation into the clinical setting. Considering the many ethical layers involved, we will first give a brief overview of the development of artificial intelligence-driven polygenic risk scores, associated ethical and social implications, challenges in artificial intelligence ethics, and finally, explore potential complexities of polygenic risk scores driven by artificial intelligence. We point out emerging complexity regarding fairness, challenges in building trust, explaining and understanding artificial intelligence and polygenic risk scores as well as regulatory uncertainties and further challenges. We strongly advocate taking a proactive approach to embedding ethics in research and implementation processes for polygenic risk scores driven by artificial intelligence.

## 1 Introduction

Machine learning (ML) techniques, in particular deep neural networks (DNNs), are increasingly being developed to generate polygenic risk scores (PRSs) using electronic health records (EHRs) as well as genomic and other health data (Ho et al., 2019; Badré et al., 2021; Elgart et al., 2022). While this may allow greater accuracy, performance and prediction ability of PRSs, it also presents a range of increasingly complex ethical challenges. PRSs are defined as “a weighted sum of the number of risk alleles an individual carries” (Lewis and Vassos, 2020). In medicine, PRSs estimate an individual’s risk of a specific condition or disease based on their genetic makeup. Even though the genomes of individuals are to a large extent similar, there are genetic differences, which are called genetic variants (Broad Institute, 2021). If a genetic variant is more common in individuals who have a specific disease, it may be associated with an increased risk of that disease (Broad Institute, 2021). A PRS takes into account all these risk variants, however minimal their effect, to estimate an individual’s risk of developing a disease (Broad Institute, 2021). Recently, PRSs have been developed to offer targeted risk prediction for a rapidly increasing number of conditions, including complex common diseases and conditions, such as breast cancer (Mavaddat et al., 2019), type 2 diabetes mellitus (Läll et al., 2017), coronary heart disease (Khera et al., 2016; Inouye et al., 2018), obesity (Khera et al., 2019), depression (Mitchell et al., 2021) and schizophrenia (Trubetskoy et al., 2022). Researchers aim to develop PRSs as a tool to prevent and more effectively treat serious diseases, disorders and conditions by identifying those at high risk who would benefit from targeted therapies.

The ethical and social implications of many PRS applications in medicine have already been widely discussed (e.g., Adeyemo et al., 2021; Knoppers et al., 2021; Slunecka et al., 2021). However, their confluence with ML has not yet been sufficiently considered in either literature or practice. We argue that the interaction between different and novel layers of ethical and social concerns pertaining to artificial intelligence (AI) and big data, as well as PRSs in research and translation into the clinical setting, stand in need of urgent consideration. This includes ethical aspects of AI as well as ethical and social implications of precision medicine and PRSs. We highlight potentially increasing complexities and the need to explore which new ethical and social issues arise from increased use of AI techniques for different PRS applications. We do so in the hope that those who aim to embed PRSs in healthcare systems take a proactive approach to embedding ethics during the research and implementation process. After giving a brief overview of the background to AI-driven PRSs, we consider the many ethical layers involved, beginning with the ethical and social implications of PRSs, then moving on to the challenges in AI ethics, and finally, exploring potential complexities of AI-driven PRSs.

## 2 Background to PRSs and AI-driven PRSs

**FIGURE 1:**
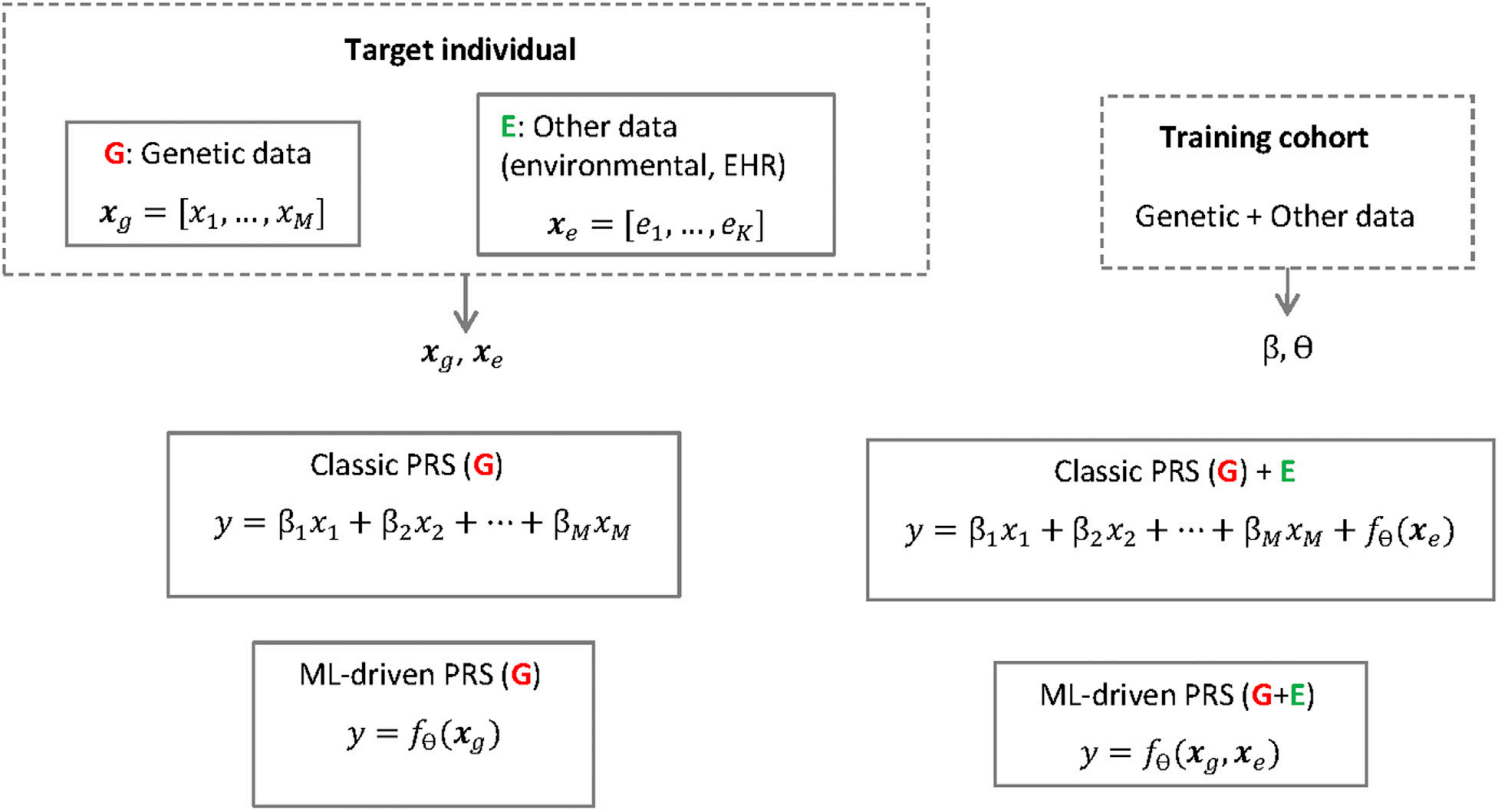
Classic PRSs and ML-driven PRSs the polygenic risk score for a target individual and phenotype of interest (y) is based on the individual's genetic data (*x*_*g*_) but can also include other data types (*x*_*e*_). The score is calculated using a linear regression (with weights *β*) or a machine learning model *f*_*θ*_ (e.g. a neural network with parameters θ). The parameters (β, θ) are learned using a separate training cohort. Note, however, that while the linear regession cofficients β are often publicly available or can be derived from published summary statistics, to train the neural network *f*_θ_ it is necessary to have access to individual level data in the training cohort.

Early studies on PRSs (Purcell et al., 2009; Dudbridge, 2013) applied the so-called *classic PRS method* (Choi et al., 2020), where the risk is calculated as a weighted sum (i.e., a linear regression) of a set of genetic risk alleles for given single nucleotide polymorphisms (SNPs) (see also Figure 1). The relevant subset of SNPs is selected using a genome-wide association study (GWAS), usually conducted in a cohort different from the target cohort, such that SNPs exceeding a certain p-value threshold are included in the calculation of the risk in the target population. Instead of using a subset corresponding to the significant SNPs, it is possible to include a much larger number of SNPs in the weighted sum to calculate the risk. When so many SNPs are included, it is necessary to prevent overfitting by applying shrinkage on the linear regression weights using either classic techniques such as the LASSO or ridge regression (Mak et al., 2017) or Bayesian methods (Ge et al., 2019), the latter having given rise to some of the most popular implementations today (Vilhjalmsson et al., 2015). The SNP weights in a PRS can be derived from effects sizes published for the GWAS cohort, where the effect of each SNP on the risk has been estimated one-SNP-at-a-time, by accounting for linkage disequilibrium (LD) between the SNPs (Choi et al., 2020). Therefore, to apply classic PRS, individual-level data are only needed for the target individuals, but not from the GWAS cohort.

Recent years have witnessed attempts to replace the linear regression in PRS calculations with more sophisticated ML methods, which promise increased accuracy due to less restrictive modeling assumptions (Ho et al., 2019; Elgart et al., 2022). For example, DNNs which belong to the broader class of deep learning (DL), have been tested in PRSs for breast cancer, leading to improved scores compared to other statistical and ML estimation methods (Badré et al., 2021). A DNN processes input SNP data by passing them successively through multiple layers, where each layer takes the features from the previous layer as input, updates them, and passes the updated features forward to the next layer. In this way, features in higher layers can represent arbitrary, non-linear combinations of SNPs instead of the simple linear summation in conventional PRSs, which may better reflect the underlying biology.

Besides applying DL to modeling the genetic component, DL can alternatively be used to extract additional predictive features from EHRs (Miotto et al., 2016), which can be combined with the genetic data as input in PRS calculation (Dias and Torkamani, 2019). For example, using non-genetic risk factors together with genetic data improves the accuracy of breast cancer (Lee et al., 2019) and coronary artery disease (Inouye et al., 2018) risk modeling with the potential to enhance risk-based screening. However, current models typically build on combining genetics and EHR features additively (i.e., a simple summation), leaving room for the development of more complete approaches, for example a DNN that takes as input the different risk factors jointly to learn about the complex interplay between them.

Current research aims to pool and assess genomic data from biobanks, cohorts or registries on an unprecedented scale by combining it with environmental, other -omics data and health data such as EHRs. Considering the increasing heterogeneity of data that is used in the development of PRSs, more complex uses of AI have also been employed, such as making use of deep phenotypic information in medical images and EHRs to support downstream genetics analyses (Dias and Torkamani, 2019). Currently, PRSs typically only involve the genetic component, which is easier to interpret. The challenges in interpretation mainly occur when other data types are included, such as EHRs or gene expression data, the latter being different from SNPs that are currently used as data for PRSs (see also Figure 1). Other such data types are likely to increase in use, so the major challenges regarding the black box nature of the DNN models will probably be more relevant in the (near) future. Although researchers aim to reveal more and more causal relations, to date, analyses with AI for PRSs are mainly limited to correlations and improving predictions, which can result in inconclusive evidence (see Section 4). Barriers to the explainability of AI for PRSs also exist due to the statistical-probabilistic properties and the difficulty of the model to uncover the more complex biological, chemical and physical mechanisms that influenced it. In addition, there is a risk of potentially superfluous or inflated correlations due to the limitations of the method through phenomena such as the recently observed “cross-trait assortative mating” (Border et al., 2022). The risks of misinterpretation of (AI-driven) PRSs by clinicians, patients and other stakeholders involved should not be underestimated, especially as there may be a risk of drawing conclusions about causal relationships too quickly and where knowledge of statistics and causality/correlation claims is too low in many groups involved. Although the difference between causation and correlation is well understood by scientists, authors point to the need for education of the public about such differentiations for PRSs (Slunecka et al., 2021).

## 3 Ethical and social implications of PRSs

The potential benefits of the clinical use of PRSs may be manifold, both for individuals and/or society: identifying individuals at risk, improving the precision and range of differential diagnoses and treatments, as well as promoting the development of intervention thresholds. Incorporating polygenic risk profiles into population screening is expected to increase efficiency in contrast to screening stratified by age (Chowdhury et al., 2013; Torkamani et al., 2018; Kopp et al., 2020), while use of combined PRSs for various conditions in healthcare systems may contribute to early identification of potential non-genetic interventions and increased life expectancy (Meisner et al., 2020). Thus, PRSs may benefit individuals and represent a dramatic improvement of public health with potential socio-economic impacts. This has led to demands by PRS advocates within the medical community for a radical rethinking of PRSs as clinical instruments that could inform clinical decisions, such as in the prioritisation of psychosocial or pharmaceutical interventions “rather than treat/not treat decisions” (Lewis and Vassos, 2020).

While they come with important benefits, discussions in the literature on the multiple ethical and social implications for the medical use of PRSs range from social and distributive justice questions to debates on scientific validity and clinical utility (Babb de Villiers et al., 2020; Lewis and Vassos, 2020; Knoppers et al., 2021; Lewis and Green, 2021; Slunecka et al., 2021; Widen et al., 2022). In the context of PRS development and clinical implementation, ethical debates reflect those on monogenic genetic findings (Lewis and Green, 2021). Common concerns relate, for example, to genetic determinism as well as the concepts of ancestry/ethnicity, where tools such as AI for risk stratification may not be representative of human diversity and whose development and use may distract attention from the social determinants of health (Lewis and Vassos, 2020; Knoppers et al., 2021; Lewis and Green, 2021). Particular concerns about the risk of genetic discrimination and eugenics are raised with regard to the application of PRSs for embryo screening (Treff et al., 2019; Tellier et al., 2021; Turley et al., 2021); most recently for pre-implantation genetic testing (PGT) (Kozlov, 2022) and premature direct-to-consumer genetic testing/genetic counselling (Docherty et al., 2021), which are also intertwined with marketability and commercialisation. Furthermore, due to underrepresentation of already underserved communities in the research process, some authors note that health disparities could increase through the use of PRSs in the clinical setting (Martin et al., 2019a).

There has been extensive discussion of the clinical and/or personal utility of PRSs (Torkamani et al., 2018; Lambert et al., 2019; Wald and Old, 2019; Lewis and Vassos, 2020; Moorthie et al., 2021; Sud et al., 2021). Scientific and clinical validity are challenges on multiple levels (Janssens, 2019; Lewis and Vassos, 2020; Knoppers et al., 2021), which touch ethical as well as epistemic concerns. PRSs, for example, do not cover the full risk for certain diseases because of the multiple factors involved. This includes e.g. environmental factors (Slunecka et al., 2021) and complex interactions between environments and PRSs (Domingue et al., 2020). Due to this complexity, interpretation of PRSs poses serious challenges, especially in relation to minors (Palk et al., 2019). From an ethical point of view, the necessity of communicating the limitations of risk prediction with PRSs therefore has to be considered in clinical applications. To this end, “effective and clear risk communication by trained professionals” should “minimize potential psychosocial effects” (Adeyemo et al., 2021). However, in this context, there is a lack of standardised PRS disclosure for individuals (Brockman et al., 2021; Lewis et al., 2022) as well as for kin, such as cascade screening for family members (Reid et al., 2021). Tools for standardisation of PRS disclosure have been developed for certain diseases, such as coronary artery disease (Widen et al., 2022), but the need for additional research on a broader range of populations and better standardisation has been emphasised (Brockman et al., 2021).

Given that PRSs are still an emerging field, there is remarkable heterogeneity around their application and reporting, thus constraining the implementation of PRSs in clinical settings (Slunecka et al., 2021). Publicly accessible catalogues and reporting standards for PRSs have been developed that are responsive to the current research landscape to allow reporting on the design and validation of PRSs within the literature (Lambert et al., 2021; Slunecka et al., 2021; Wand et al., 2021), such as the NHGRI-EBI, an extensive database of summary statistics of GWAS (Buniello et al., 2018). One aim of these efforts is to generate comparable PRSs metrics of performance (Lambert et al., 2021). This should increase the reproducibility and transparency of the PRS development process as well as support studies evaluating the clinical utility of the respective PRSs (Lambert et al., 2021). External and systematic PRS studies with benchmarking should also contribute to these aims (Wand et al., 2021). Another practical ethical issue is that the application of PRSs for medical purposes is presently uncertain under the majority of legal frameworks (Lewis and Vassos, 2020; Adeyemo et al., 2021).

Moreover, some authors also point out the importance of seeing PRSs in the respective context (Chatterjee et al., 2016; Torkamani et al., 2018; Slunecka et al., 2021), considering that the scope and diversity of available data (for instance, ancestry) and the techniques used to produce and use the scores are continuously changing (Trubetskoy et al., 2022). This therefore necessitates consideration, e.g., of the particular PRSs and the disease for which the PRSs were designed and the sophistication of the PRS itself. Consequently, the ethical and social implications need to be explored, taking into account the respective context. For example, specific ethical concerns in PRSs have been increasingly described for psychiatric conditions from informational risks in the use of the PRS in clinical setting, to the research showing links between the condition and social factors such as socioeconomic status or potential use in prenatal testing among others (Agerbo et al., 2015; Loh et al., 2015; Martin et al., 2019b; Palk et al., 2019; Docherty et al., 2021; Murray et al., 2021). This may differ for other conditions, for instance, in terms of actionability or potential for stigmatisation.

## 4 Challenges in AI ethics

There is much debate on ethical aspects around AI in healthcare (Morley et al., 2020), the role that AI should play (Rigby, 2019), the role and ethical implications of “explainability for AI in healthcare” (Amann et al., 2020), and ethical challenges of ML (Vayena et al., 2018) and of DL in healthcare (Char et al., 2018; Miotto et al., 2018). In particular, the following ethical and social challenges are often discussed in AI ethics (Mittelstadt et al., 2016; Floridi et al., 2021; Tsamados et al., 2021): How to ensure fairness and justice, overcome biases, ensure explainability, transparency, traceability, accountability, privacy, confidentiality, data protection and patient safety–how to design AI for the common good.

In AI ethics, not only are there normative concerns about algorithms such as “unfair outcomes” and “transformative effects”, but also epistemic concerns such as “inconclusive evidence”, “inscrutable evidence” and “misguided evidence” (Mittelstadt et al., 2016; Tsamados et al., 2021), and often epistemic and normative concerns come together as in the case of traceability. Issues such as the black box problem, accountability and transparency can be subsumed under inscrutable evidence (Mittelstadt et al., 2016). The black box problem in ML hinges on the lack of explainability as to how results are generated. The importance of this is also reflected in European law like the EU General Data Protection Regulation (GDPR) (European Parliament and Council of the European Union, 2016), which entails a general “right to explanation” (Goodman and Flaxman, 2017) for users and a future where explainability could become a legal requirement for ML specifically. The proposed Artificial Intelligence Act of July 2021 explicitly includes the requirement that AI systems be explainable for high-risk sectors (European Commission, 2021). The literature in recent years has repeatedly underlined the need for explainable AI (xAI) in medicine (Ribeiro et al., 2016; Hudec et al., 2018; Holzinger et al., 2019; Azodi et al., 2020), which is seen as (part of) a possible solution to many of the above-mentioned challenges in AI applications in healthcare.

Inconclusive evidence (Mittelstadt et al., 2016) involves ethical issues of causality and correlation, probabilities and predictions. Inconclusive evidence and incorrect causal associations and correlations are a problem for any statistical model, which can be the result, e.g. of biased sampling or hidden contamination. Authors generally point to the need to understand causality of the representations in ML systems (Pearl, 2009; Gershman et al., 2015; Peters et al., 2017; Holzinger et al., 2019). Furthermore, as substructures from genomic and population data are correlated, this can potentially result in false causal associations (Sohail et al., 2019) and misleading information based on bias embedded in genomic data (see Section 5.1). Increasing the robustness of the detected effects across different populations would go some way towards separating true causal effects from spurious associations. In genetics, replicating the findings in multiple cohorts is usually a stipulation, but more work is required to ensure inclusion of more diverse populations (see Section 5.1).

The topic of “misguided evidence leading to bias” (Mittelstadt et al., 2016) and “unfair outcomes leading to discrimination“ (Mittelstadt et al., 2016) are key issues in AI ethics. In medical AI, biases (Obermeyer et al., 2019) abound, and the replication of biases and the amplification of real-world injustices by algorithms poses a serious risk.

There are many different proposals for frameworks on how the challenges of applying AI in medicine should be addressed ethically, which principles and values are of particular importance and which guidelines should be followed. Ethical challenges exist in terms of principles, not only regarding which principles should be considered crucial, but also in terms of differences in what the principles mean, e.g. what justice encompasses, as there are many different forms of justice derived from different philosophical theories and different underlying values (Whittlestone et al., 2019). Furthermore, there is the question of what “for the good of society” means—What would AI that is focused on the common good look like? This would need to be discussed and defined in each context (Whittlestone et al., 2019).

Another challenge usually arises when principles conflict with each other, as is often the case with AI in healthcare. Explainability is often not technically possible, and the benefits of AI can vary in significance, so the trade-off would have to be weighed up for each AI system and context. Another major ethical challenge around AI is putting principles into practice. Authors point out that attention needs to be paid to the tensions and conflicts that arise in this process and that these need to be addressed (Whittlestone et al., 2019) so that risks can be avoided and the benefits of AI can be reaped.

## 5 Bringing ethical and social aspects of PRSs and AI ethics together—New complexities for AI-driven PRSs?

In bringing ethical and social implications of PRSs and of AI ethics together, we would like to point out potential new complexities for AI-driven PRSs. Particularly around the following topic clusters which will be discussed in detail in what follows.

1) More complexity regarding fairness and justice
2) Challenges in building trust, communication and education
3) Privacy and autonomy challenges
4) Regulatory uncertainties and further challenges

### 5.1 More complexity regarding fairness and justice

Although many researchers point out the opportunities of xAI and interpretable ML (iML), two ethically relevant issues with respect to explanatory methods remain generally difficult to solve: different biases within datasets leading to biased DNN and suspicion of bias in results leading to unfairness (Ras et al., 2018). This could apply also to ML application for PRSs on multiple levels: many biases in PRS development can be linked to biases in the combination of EHRs with genomic and further health data as well as in the substructures of this data.

Firstly, the majority of genetic studies lack diversity (Sirugo et al., 2019). PRSs have mainly been developed with datasets from European populations and predictions of genetic risk are susceptible to unequal outputs (performance levels) across different populations as they are underrepresented in training data, which hinders generalisability (Martin et al., 2019a). Authors observe that research infrastructures like biobanks may suffer from “recruitment bias” as a risk which “infringes on the principle of justice, influences representativity of biobank collections and has implications for the generalizability of research results and ability to reach full statistical power” (Akyüz et al., 2021).

Secondly, further data biases can be linked to many other factors. There is a considerable gap in medical studies on the representation of women (Daitch et al., 2022) as the case of cardiovascular disease also shows (Burgess, 2022). More broadly, gender bias can be found in written documents used for certain ML techniques (Bolukbasi et al., 2016). Gender bias may also occur when heteronormative paradigms are not met, e.g., when data on gender and sex do not match and are therefore automatically excluded for analysis, which is currently a common practice in genomics (American Medical Association, 2018; Ganna et al., 2019). EHRs can contain multiple biases resulting e.g., from physician bias or certain delivery of care (Ching et al., 2018; Gianfrancesco et al., 2018), and even laboratory measurements (which are considered less biased) can show bias resulting from the patient health state and healthcare process (Pivovarov et al., 2014)—although they may be representative regarding population (Kerminen et al., 2019; Adeyemo et al., 2021). Overall, there are substructures in genomics and other health data that can be linked to actual differential causal relationships between health outcomes and putative risk factors. Other substructures can be traced to external factors such as cultural practices, socioeconomic status and other non-causal factors that relate to healthcare provision, access to medicine and clinical trials (Gianfrancesco et al., 2018; Dias and Torkamani, 2019; Sirugo et al., 2019).

Apart from the bias in data, *machine bias* has to be mentioned in the context of AI use for PRSs. This encompasses the biases that are learned by the models (Ching et al., 2018; Dias and Torkamani, 2019). In this context, one criterion for iML for genetic risk prediction could be whether a certain model is adequately interpretable for bias to be detected (Ching et al., 2018; Dias and Torkamani, 2019). Authors call for standards of fairness in order to diminish disparities caused by bias of ML in genetic risk prediction (Dias and Torkamani, 2019; McInnes et al., 2021). Moreover, they point to the necessity for careful application of AI and differentiation between the various forms of bias arising when AI is applied to genetic risk prediction (Dias and Torkamani, 2019). Tools are already being developed to help eliminate machine bias. This is not only intended to eliminate bias of ML, but also to create diagnostic systems that are much freer from human bias than classical diagnostics by physicians allow (Shen et al., 2019). These and further innovative sorts of techniques should also be consistently considered for ML use for PRSs.

In addition to injustice due to biases, *injustice and unfairness regarding data access and sharing data and algorithms* is also an issue for AI-driven PRSs. In this regard, biased processes and results are co-produced, potentially sustaining existing inequalities and unfairnesses. Further, apart from comprehensibility, accessibility can be considered as the second main component of transparency in generating information about how algorithms function (Mittelstadt et al., 2016). While many advances have been made thanks to international initiatives and large interdisciplinary research consortia, authors still highlight the ongoing need to collect, harmonise and share data in genomics and healthcare (Diao et al., 2018; Lambert et al., 2021). The Polygenic Risk Score Task Force of the International Common Disease Alliance has called for the “GWAS research community, global biobank collaborations, and private direct-to-consumer companies” (Adeyemo et al., 2021) to create requirements for public sharing of summary statistics using standardised formats, with the aim of avoiding the exacerbation of worldwide health inequalities (Adeyemo et al., 2021). However, sharing DL models with the biomedical data and health records of individuals not only faces legal and technical barriers but also poses a major “cultural challenge” (Ching et al., 2018). A culture that rewards discovery rather than the production of data will have a difficult time motivating researchers to share their hard-earned datasets (Ching et al., 2018). However, as is pointed out in recent articles, it is this data that would drive DL (Ching et al., 2018).

Apart from well-known privacy regulations and standards in medical and biological research (Ching et al., 2018), factors such as the costs related to regulations for medical devices may also play an important role in access to PRSs, creating inequalities among populations, subgroups and countries (Adeyemo et al., 2021). Not only does global cooperation contribute to more equity in medical research and healthcare, it also serves an important role for the improvement of clinical validity and utility of PRSs (Adeyemo et al., 2021; Knoppers et al., 2021). Moreover, an open exchange of AI models for genetic risk prediction with the medical and scientific communities is called for to enhance transparency, where the model sharing should include details such as model weights, source codes and meta diagrams (Dias and Torkamani, 2019). Synthetic genetic and phenotypic data (Abadi et al., 2016) is suggested for genomic projects (Moorthie et al., 2021) and is already being tested in PRS development to provide greater diversity in genetic data, avoid biases and privacy issues. Furthermore, protecting data and privacy are very relevant for public-private partnerships (Murdoch, 2021), which play an increasingly important role for the implementation and dissemination of PRSs.

### 5.2 Challenges in building trust, communication and education

One of the greatest challenges in translating PRSs to the clinical setting is the communication of PRSs. This includes communication to and dialogues with the public(s) and patients as well as educating all other stakeholders involved. The challenge of communicating PRSs in the clinical setting, particularly for doctors (Fiske et al., 2019), is magnified when explaining AI-driven PRSs.

In general, we highlight the need for reflection on epistemological questions around AI use for PRSs and the corresponding normative aspects. It is important to ask what it means to explain, interpret and understand AI-driven PRSs. This should ideally incorporate different perspectives for certain stakeholders and involve further associated questions, e.g., what researchers consider an explanation to be, what kind of explanation users want and need (Slunecka et al., 2021) and what criteria are relevant for explainable PRSs. With the advance of xAI and iML, it is also worth considering how much/what kind of explainability is required for the clinical application of PRSs and how much/what kind of interpretability is clinically meaningful.

With regard to the literature reviewed and the existence of different definitions of explainability, explicability, interpretability and comprehensibility in scientific teams and clinical settings, we argue that awareness of these differences of terms must be raised both in scientific publications and in practice. This would also have the ultimate goal of improving the explainability of the risk scores and the underlying AI mechanisms.

Stakeholders in research and development as well as healthcare areas are constrained to consider the uncertainty of AI-generated PRS predictions and thus need to develop means of dealing with them in a structured, transparent and responsible way. Even if a more explainable ML for PRSs is developed, the question of how to communicate and generally deal with uncertainty due to lack of explainability of ML for PRSs nevertheless requires discussion and translation into appropriate standards. Embedded ethics approaches (McLennan et al., 2022) in both the research and clinical settings could help resolve the challenge of detecting and reflecting on ethical issues as well as communicating them.

Regarding communication of AI-driven PRSs, there is a clear need for engagement with technical, medical and ethical aspects of PRSs and AI for all the different stakeholders involved. We strongly recommend adopting interactive/participatory engagement practices (Horst et al., 2017), especially between clinicians and patients for AI-driven PRSs. This means limiting or avoiding deficit models of communication, i.e., unchallengeable, non-reflexive (Wynne, 1993) communication, which sees audiences (including any actors other than experts) as deficient both in knowledge and capacity to comprehend (Bell et al., 2008). In light of the developments in e-health, citizen-patients are not considered passive recipients of information, but rather self-informing, active individuals (Felt et al., 2009). Furthermore, the respective educational, socioeconomic and cultural background of individual patients and their families has to be considered when, for example, physicians explain PRSs (Slunecka et al., 2021).

In general, one of the biggest challenges of AI-driven PRSs today is trust in AI/AI-driven PRSs and trust in the medical institutions that will use these technologies on a large scale. However, there is a lack of specificity in the literature on issues of trust in the recently developed AI-driven PRSs. This represents a future issue that will need to be addressed with interdisciplinary teams.

Problems of AI explainability add complexity to matters of trust for AI-driven PRSs. Lack of transparency and lack of human understanding of AI black boxes raises the question of how all kinds of end-users create their relationship with AI. Scholars emphasise the importance of explainable AI (Holzinger et al., 2019) and DL models in medicine by arguing for the trust-building effect they have (Ribeiro et al., 2016). They point to the importance of understanding the rationale underlying the predictions of ML modelling when evaluating trust, which is considered crucial for decisions on the use of new models and actions based on predictions (Ribeiro et al., 2016). Interpretability is reflected in the “fidelity-interpretability trade-off” (Ribeiro et al., 2016) and is key to building trust in AI among healthcare professionals. Practitioners are very unlikely to accept a DL system that they do not understand (Miotto et al., 2018). It is noted that the interpretability of the model in genomics is critical to convincing health professionals of the validity of the actions the prediction system recommends, e.g., to explain which phenotypes drive certain predictions (Miotto et al., 2018).

The High-Level Expert Group on AI of the European Commission proposes trust as one of the defining principles for their AI ethics guidelines (High-Level Expert Group on AI, 2019). However, the technical solutions to the issue of trust, as discussed above, are unlikely to become available in definitive form. We therefore suggest that the social and relational considerations are paramount if we are to create a workable framework for establishing trust. This means the question of how trust is built needs to be addressed by adopting a more reflexive and interdisciplinary perspective. This also includes discussion of the *trustworthiness* of AI use for PRSs. Which is to say, discussions about dependable, trustworthy ML use for the PRSs and what requirements and criteria should be placed on the trustworthiness of AI for PRSs must perforce address contextual questions, such as what trust means in a particular situation or context. The FUTURE-AI initiative has created dynamic best practices for trustworthy AI in healthcare (Future AI, 2022). Empirical and theoretical studies on the ethical and social issues of AI-driven PRSs and trustworthiness are needed so that this knowledge can then be integrated into the development and application of AI-driven PRSs.

Overall, we recognise that education and training of AI-driven PRSs would need to cover tech/AI literacy, risk interpretation/statistical knowledge, genomics/PRS knowledge, communication skills and ethical reflection skills of the stakeholders involved—of course with different granularities depending on the stakeholders: patients, relatives of patients, various public(s), healthcare professionals, medical/nursing students, researchers, technicians, ethics committees, clinical ethics teams, business partners and all the other stakeholders involved in research and development as well as the translation, implementation and application of AI-driven PRSs.

For AI in medicine generally, there is a need to increase education and training for different stakeholders in the healthcare system on applications of technology driven by data (Meskó et al., 2017; Xu et al., 2019). For ML in genomics, authors stress the need to bridge the gaps regarding clinical knowledge and interpreting models (Diao et al., 2018). Others consider the training of clinical staff to be a major challenge for the implementation of PRSs in the clinical setting (Torkamani et al., 2018; Slunecka et al., 2021). The unique nuances of PRSs and GWAS development are mostly unfamiliar to clinicians at this point (Martin et al., 2019a). Concrete suggestions have been made for enhancing education about PRSs for inclusion in the regular curriculum for medical students and in the ongoing education for medical professionals, covering the limitations of PRSs and different forms of risk (Slunecka et al., 2021). In addition, there are different proposals for how experts in genetic risk assessments could be involved in the clinical setting. Furthermore, education of the public(s) is crucial in implementing PRSs for public screening. The website of the National Human Genome Research Institute of the National Institutes of Health (UK), for instance, aims to explain to the public how PRSs work and how to interpret them. Apart from that, sensitivity, reflection and discussion on relationality and power relations of patients, doctors, healthcare and research institutions as well as biotechnology/genomics companies are important issues in the development of AI-driven PRSs. Based on a renewed understanding of how citizens engage with physicians and information technologies in health setting (Felt et al., 2009), empowering citizens and patients is among the key developments for the application of AI-driven PRSs.

### 5.3 Privacy and autonomy challenges

When large amounts of genomic data and EHRs are used to generate PRSs with AI, privacy is a key issue. A crunch question is whether protection of personal/patient data trumps transparency and right of access to data or *vice versa*. There are also multiple questions revolving around the extent to which anonymisation can be ensured with the large amounts of data used for PRSs, new AI technologies and what informed consent should look like for different uses of PRSs driven by them. For example, the differential privacy method, in which noise is added to data to prevent revealing individual information in case summaries of the data were to be published, does not scale easily to high-dimensional genetic data (Roth and Dwork, 2013). While there are efforts in medicine and PRS development aimed at protecting privacy (Abadi et al., 2016; Simmons et al., 2016; Ching et al., 2018; Beaulieu-Jones et al., 2019; Zhang et al., 2021), it is unclear how these could be implemented or policed on a large scale for AI-driven PRSs. Despite the discourse of exceptionalism of big data research, privacy is still an issue that is tightly entangled with autonomy (Rothstein, 2015). However, in a data-rich environment, genomic data, which is by definition shared in differing amounts with biological relatives, poses further challenges to our understandings and practices of privacy and autonomy, but also anonymisation or risk of genomic identifiability, raising the necessity for a “post-identifiability” lens (Akyüz et al., 2023). Thus, privacy and autonomy are challenges in their own right due to the peculiarity of genomic data.

### 5.4 Regulatory uncertainties and further challenges

As for healthcare in general, the need for complementary measures to explainability such as regulation (Markus et al., 2021), enhancing the quality of healthcare data for DL (Miotto et al., 2018) and external validation (Markus et al., 2021) have to be considered for AI-driven PRSs. The need for regulatory measures for PRSs in general is highlighted in the literature reviewed (Adeyemo et al., 2021; Knoppers et al., 2021; Slunecka et al., 2021). Standardisation of regulation frameworks for PRSs as medical devices (Adeyemo et al., 2021) is urgently required. With AI-driven PRSs, it is even more important to establish internationally standardised regulation frameworks which are responsive to the dynamic and fast-evolving technical and scientific findings around PRSs. Flexible, on-demand “*ad hoc*” guidance to positively enhance ongoing algorithm improvement (Vayena et al., 2018; Dias and Torkamani, 2019) would support the ethically sound development of AI-driven PRSs. However, regulatory measures can be a burden for people with access to PRS technology (Knoppers et al., 2021). In this sense, the challenge of creating a balance between sufficient regulation and rapid scientific advancement in the application of AI for PRSs must be considered in the development of AI-driven PRSs.

Beyond the ethical concerns mentioned above, further ethical challenges of AI-driven PRSs, such as informed consent procedures for AI-driven PRSs in absentia of explainability could become even more relevant in the future. In addition, the importance of AI for ethics committees has to be emphasised as does the need to involve research ethics committees and clinical ethics committees in the translation and implementation of AI-driven PRSs.

## 6 Conclusion

Our article has delineated the multiple layers of ethical and social concerns associated with PRSs, AI for PRSs and AI-driven PRSs in medicine. A clear limitation of most ML-based approaches compared with the *classic PRS* method is the requirement for individual level data to train the models, whereas the latter uses publicly available summary statistics about estimated effect sizes. Hence, there is room for development of new ways to leverage published summary statistics in training of more flexible ML-based PRS methods. Another limitation and future challenge common to all PRS methods is the poor generalisability of the scores in populations with different ancestries, which also stems from different allele frequencies, linkage disequilibrium and genetic effect sizes in different populations (Wang et al., 2022). Regarding the use of AI in PRS, there is great potential for improvement by developing models that integrate a variety of health data types and risk factors into comprehensive predictors of disease risk (Dias and Torkamani, 2019). The clinical utility of PRSs is currently hotly debated; thus, more research is warranted on the best ways to implement PRSs as part of clinical practice, either to improve diagnoses, personalise treatments, or as part of preventive medicine (Torkamani et al., 2018; Choi et al., 2020). In particular, the additional challenges for the clinical implementation posed by the AI based PRS methods remain to be addressed. Furthermore, our discussion of some of the ethical issues that need to be considered in AI-driven PRS is in no way exhaustive. Rather, this article can serve as a basis for further discussions of the ethical challenges that could arise from the future application of AI-driven PRSs.

Where PRSs, ML and big data are part of the picture, we have teased out the more complex ethical challenges emerging from the relation between them, as well as pertaining to them individually. Based on a comprehensive review of the existing literature, we argue that this stands in need of urgent consideration for research and translation into the clinical setting. Different layers of ethical implications could lead to more challenges for explainability of AI-driven PRSs, more complexity of fairness with biases in data (sets) and ML for PRSs and biased outputs, more challenges in building trust, communication and education as well as regulatory uncertainties for and challenges in privacy and autonomy of AI-driven PRSs. Among these, we would especially like to highlight a lack of specificity in the literature on issues of trust in the more recent instantiations of AI-driven PRSs. We maintain that this is a future challenge that will need to be addressed in interdisciplinary, multi-stakeholder teams. The fact that the lack of explainability seems to be an inherent problem of certain ML techniques, which may never be fully solved, should not hinder efforts to make ML for PRSs more explainable and trustworthy for all stakeholders involved in the healthcare system. It has become clear that much of the more explainable PRSs depends not only on more explainable ML techniques, but also on awareness, context- and user-specific communication and engagement, education and training for all stakeholders. In addition, there are limitations to the influence of explainable ML that relate to ethical and social aspects associated with large amounts of data, such as EHRs, genomic and other health data fed into ML models. Apart from more technical research on e.g. techniques of explainable ML for PRSs, more ethical analyses are needed, covering epistemic and normative aspects of AI-driven PRSs including methods of normative and empirical ethics. We have also pointed out that hitherto there are few to no regulatory guidelines, and a lack of commensurate up-to-date research, let alone clear advice on how to communicate the potential implications, costs or benefits of these technological advances to and between the various stakeholders involved. For this, technical and bioethical content as well as discussions on the larger societal implications and public health aspects should also be included in the training for students and healthcare professionals. Although there are efforts to address the ethical and regulatory challenges of AI-driven PRSs, more work is required when AI tools are used with more complex health data such as EHRs and medical images or real world data. This should be an important item on the agenda of citizens, policymakers, scientists and funders of AI-driven PRS development as a co-production. This approach would make an important contribution to the clinical utility of PRSs in terms of transparency, responsibility and finally trustworthiness.

If we fail to address these challenges, the danger is that not only will advances in AI and/or the applications of PRSs outstrip our ability to understand or regulate them, but that the potential for overreliance and indeed misapplication or misuse from an ethical and social standpoint may create further and insurmountable complexities in the future.

## Data Availability

The original contributions presented in the study are included in the article/supplementary material, further inquiries can be directed to the corresponding author.
